# Clinical-radiologic-pathologic characterization of diabetic mastopathy: an analysis of 21 cases

**DOI:** 10.3389/fendo.2026.1691578

**Published:** 2026-01-21

**Authors:** Juan Chen, Chao Zhang, Zhilong Liu, Zhuojun Qi, Lele Song

**Affiliations:** 1 Department of Ultrasonography, Shanxi Provincial People’s Hospital, Taiyuan, China; 2 Department of Ultrasonography, Shanxi Provincial Cancer Hospital, Taiyuan, China; 3 Department of Interventional Ultrasonography, First Hospital of Shanxi Medical University, Taiyuan, China

**Keywords:** breast, diabetic mastopathy, diagnosis, mammography, ultrasound

## Abstract

**Objective:**

To characterize the clinic-radiologic features of diabetic mastopathy (DMP), with a focus on distinctive ultrasound findings that may aid in differential diagnosis and reduce unnecessary interventions.

**Methods:**

A retrospective multicenter analysis was performed on 21 pathologically confirmed DMP lesions from 17 patients (2014–2024). Mammographic and ultrasonographic features were independently assessed by two radiologists according to the Breast Imaging Reporting and Data System (BI-RADS, 5th edition). Clinical data including diabetes history were reviewed.

**Results:**

The median patient age was 58 years, with a median diabetes duration of 23.3 years. Most patients presented with a palpable mass (94.1%). Mammography showed probably benign findings (BI-RADS 3) in 73.7% of lesions, with an additional 10.5% assessed as benign (BI-RADS 2). In contrast, ultrasound revealed suspicious features: all mass-forming lesions had irregular margins (100%), 89.5% showed posterior shadowing, and critically, 78.9% demonstrated an infiltrative growth pattern with an absence of space-occupying effect. One atypical case presented with bilateral ductal dilation. Core needle biopsy yielded a specific DMP diagnosis in only 68.4% of lesions. Surgical excision was performed in 76.2% of lesions, with recurrence in 23.1% during a median follow-up of 33.5 months.

**Conclusion:**

DMP presents a diagnostic paradox of benign mammographic but suspicious sonographic appearances. The characteristic “absence of space-occupying effect” on ultrasound is a key differentiating feature from breast carcinoma. Recognizing this sign, along with atypical patterns like ductal dilation, can increase pre-biopsy diagnostic suspicion in patients with long-standing diabetes, potentially guiding more conservative management. Histopathological confirmation remains essential.

## 1 Introduction

Diabetic mastopathy (DMP) is a rare benign breast lesion marked primarily by stromal fibrosis and sclerosis (1). This non-suppurative inflammatory condition is typically found in patients with long-standing diabetes mellitus (2, 3). First described by Soler and Khardori in 1984 (4), DMP was initially reported in 12 cases of type 1 diabetic patients presenting with breast masses, where histopathological examination revealed stromal fibrosis and lymphocytic infiltration. Subsequent studies have confirmed its occurrence in type 2 diabetic patients as well (5). The incidence of DMP is relatively low, accounting for approximately 1% of all benign breast lesions (6). Owing to its distinct histopathological appearance, it is also termed sclerosing lymphocytic lobulitis in the literature.

Clinically, DMP often presents as a painless, firm, and poorly mobile mass. Diagnosis remains challenging because mammographic findings are frequently nonspecific, whereas sonographic features can closely mimic those of breast malignancy. This imaging overlap not only leads to diagnostic uncertainty but also often results in unnecessary invasive procedures—including core needle biopsy or even surgical excision—to establish a definitive diagnosis. The pathological features of DMP evolve with disease progression (7, 8). Early stages exhibit marked lobular hyperplasia accompanied by extensive periductal and intralobular lymphocytic infiltration and mild fibroconnective tissue proliferation. In advanced stages, breast lobules may show significant atrophy or even disappearance, along with prominent stromal fibroconnective tissue hyperplasia, hyalinization, increased lymphocytic infiltration forming lymphoid follicles, and the presence of numerous epithelioid fibroblasts within the stroma.

This study retrospectively analyzed the clinical, mammographic, and ultrasonographic characteristics of 21 DMP lesions, aiming to elucidate their distinguishing features and improve diagnostic accuracy. Given the benign yet clinically deceptive nature of DMP, enhancing its differentiation from breast cancer is essential to guide appropriate management and reduce the risk of overtreatment.

## 2 Material and methods

### 2.1 Ethical approval

This retrospective study was reviewed and approved by the Institutional Review Board (IRB) of Shanxi Provincial People’s Hospital. Due to the retrospective nature of the study and the use of anonymized data, the requirement for informed consent was waived by the IRB.

### 2.2 Study population and case identification

A retrospective review was performed on the pathology databases (2014–2024) of three tertiary hospitals in Shanxi Province (Shanxi Provincial People’s Hospital, First Hospital of Shanxi Medical University, and Shanxi Provincial Cancer Hospital). The databases were searched using a combination of the following keywords: “diabetic mastopathy”, “sclerosing lymphocytic lobulitis”, “lymphocytic mastitis”, “fibrous disease of the breast”, and “diabetic breast disease”.

All retrieved pathology reports were independently screened by two researchers. Inclusion criteria were: a pathology report with a definitive diagnosis of either “diabetic mastopathy” or “sclerosing lymphocytic lobulitis.” Exclusion criteria included: (1) pathology reports containing only descriptive diagnoses (e.g., “breast fibrosis with lymphocytic infiltration,” “chronic mastitis”) without a conclusive link to DMP and lacking supportive immunohistochemistry or clinical correlation; (2) incomplete clinical data. Based on these criteria, a total of 82 pathology records were excluded due to descriptive-only pathology reports that lacked a definitive diagnosis of DMP. Ultimately, 21 pathologically verified lesions (from 17 patients) were included in the final study cohort.

### 2.3 Mammography and ultrasonography

Among the 17 patients, 15 underwent mammography using GE Senograph 2000D and Hologic Lorad Selenia digital mammography systems. Standard craniocaudal (CC) and mediolateral oblique (MLO) views were obtained to evaluate lesion location, size, margins, calcifications, abnormal vascularity, and axillary lymph nodes.

Additionally, all patients underwent ultrasonography. The following ultrasound systems were used: GE LOGIQ E9 with an ML6–15 MHz transducer, HITACHI Aloka ARIETTA 70 with an L441 transducer,

Supersonic Aixplorer with an SL15–4 transducer, Siemens Acuson Sequoia with an 18L6 transducer, Philips EPIQ 5 with an eL18–4 transducer.

Patients were placed in the supine position, with both breasts and axillae fully exposed. Radial scanning was performed in a clockwise manner centered on the nipple to assess lesion location, size, echogenicity, margins, calcifications, posterior acoustic shadowing, vascularity, and axillary lymph node status. The sonographic feature “absence of a space-occupying effect” was specifically evaluated. It was defined as the lack of displacement, compression, or bulging distortion of the surrounding normal breast parenchyma and Cooper’s ligaments by the lesion. During scanning, this feature was assessed through dynamic multi-planar observation, confirming an “infiltrative” or “blending” growth pattern of the lesion with the surrounding tissues, rather than an “expansile” mass effect.

### 2.4 Ultrasound-guided core needle biopsy

CNBs were performed with a 14-gauge automated core biopsy needle (22-mm throw; Bard^®^ Magnum^®^ Reusable Core Biopsy System, Bard Medical, Tempe, AZ, USA). The retrospective pathological reports were reviewed by a pathologist with 10 years of breast pathology experience.

### 2.5 Image interpretation and clinical data collection

Image interpretation was performed independently by two radiologists (Dr. Chen and Dr. Zhang, with 8 and 20 years of experience in breast imaging, respectively). Both radiologists were blinded to the final pathological results during the initial imaging review. Each radiologist independently evaluated all mammographic and sonographic images, assessing features including lesion location, size, margins, echotexture, posterior acoustic features, calcifications, and vascularity, and assigned a category according to the Breast Imaging Reporting and Data System (BI-RADS, 5th edition) guidelines (9).

Following the independent reviews, any discrepancies in BI-RADS assessment or key feature description were resolved through a consensus discussion between the two radiologists, with reference to the BI-RADS atlas. A final agreed-upon assessment was recorded for each lesion.

A formal quantitative assessment of inter-reader agreement (e.g., using kappa statistics) was not performed due to the retrospective study design and the primary goal of establishing a consensus diagnosis for clinical correlation. This represents a methodological limitation of our study.

Clinical information was obtained by reviewing medical records, including chief complaints, symptoms, and diabetes history. The diagnosis and classification of diabetes mellitus—type 1 (T1DM) versus type 2 (T2DM)—for each patient were retrospectively confirmed based on the following information documented in the medical records: clinical diagnosis, age at onset, treatment regimen (specifically, insulin dependence from diagnosis for type 1 diabetes mellitus, T1DM), and relevant laboratory findings, for example, the presence of autoantibodies such as glutamic acid decarboxylase 65-kDa antibody (GAD65) in T1DM, when available. Data regarding glycated hemoglobin A1c (HbA1c) levels and the presence of diabetes-related complications were also extracted from the records when documented; the absence of such data for some patients is acknowledged as a limitation. Missing data were classified as “unknown” and excluded from analysis.

### 2.6 Statistical analysis

Given the exploratory and descriptive nature of this study and the relatively small sample size (21 lesions from 17 patients), the data are summarized using descriptive statistics. Continuous variables are presented as median (range) or mean ± standard deviation, as appropriate, and categorical variables are expressed as frequencies (percentages). Formal inferential statistical tests or correlation analyses (e.g., between diabetes duration and imaging patterns) were not performed. This approach was chosen to avoid overinterpreting the findings from a limited cohort and to provide a clear, foundational description of the clinicopathologic and imaging spectrum of DMP, which remains underreported. All analyses were performed using SPSS (version 26).

## 3 Results

### 3.1 Clinical characteristics

This study included a total of 17 patients with pathologically confirmed DMP, comprising 21 lesions, of which 4 patients (23.5%) had bilateral involvement. The clinical characteristics of the 17 patients at initial presentation are summarized in **Table 1**. All patients were female with a median age of 58 years (range: 43–68 years). The majority (16/17, 94.1%) presented with a palpable breast mass, while one patient (5.9%) exhibited nipple discharge as the primary symptom. Diabetes mellitus history revealed type 1 DM in 5 cases (29.4%) and type 2 DM in 11 cases (64.7%), with one case (5.9%) of unspecified type. The median diabetes duration prior to DMP diagnosis was 23.3 years (range: 5–49 years). Treatment regimens included insulin therapy (10 cases, 58.8%) and oral hypoglycemic agents (3 cases, 17.6%), while 4 cases (23.5%) had undocumented treatment. Diabetes-related complications were documented in 7 patients (41.2%), with unavailable data for the remaining cases.

**Table 1:** Clinical characteristics of 17 DMP patients.

Characteristic	Number (%)
Chief complaint	
Palpable mass	16, 94.1%
Nipple discharge	1, 5.9%
Diabetes type	
Type 1 DM	5, 29.4%
Type 2 DM	11, 64.7%
Unknown	1, 5.9%
Diabetes treatment	
Oral hypoglycemic agents	3, 17.6%
Insulin-dependent	10, 58.8%
Unknown	4, 23.5%
Diabetes complications	
Present	7, 41.2%
Unknown	10, 58.8%

DMP, diabetic mastopathy; DM, diabetes mellitus.

### 3.2 Mammographic characteristics

In this study, 19 out of 21 DMP lesions underwent mammography. Two lesions (10.5%) were categorized as BI-RADS 0 due to focal glandular thickening and increased density, which obscured clear assessment. One lesion, located in the right breast, presented as a large (approximately 72 × 64 mm), high-density mass with smooth and well-defined margins, lacking spiculated borders. Given its considerable size, it was classified as BI-RADS 4b. Two lesions (10.5%) were assessed as benign (BI-RADS 2) due to the presence of coarse calcifications visible bilaterally. The remaining 14 lesions (73.7%) demonstrated dense glandular tissue without any suspicious features (e.g., mass, microcalcifications, distortion) and were therefore classified as probably benign (BI-RADS 3).

### 3.3 Ultrasonographic features

**Figure 1:**
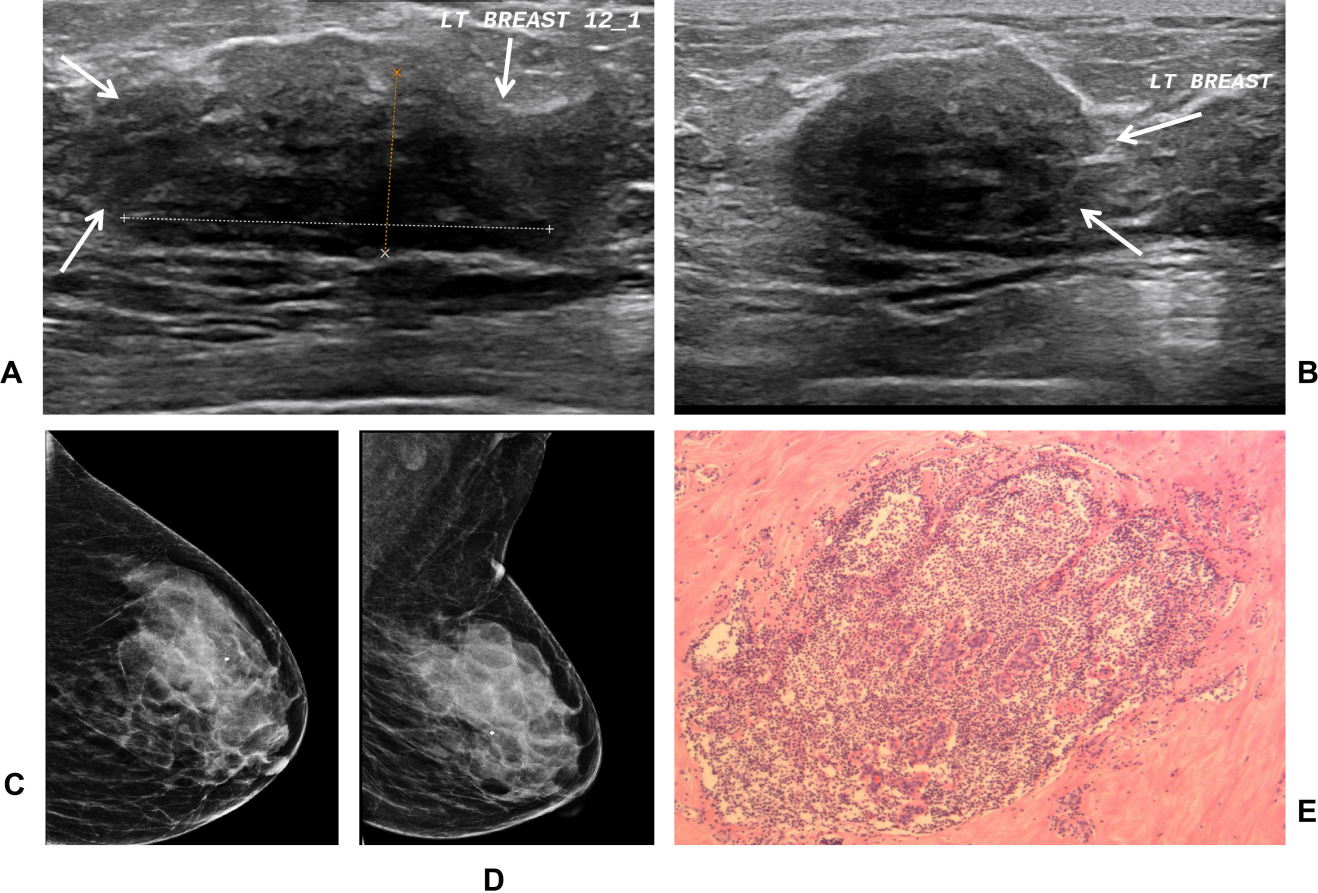
A 68-year-old woman (Patient 1, **Table 3**) with 20-year history of type 2 diabetes mellitus (10 years insulin therapy) presenting with a left breast mass. **(A, B)** Transverse **(A)** and longitudinal **(B)** ultrasound images show a heterogeneous, ill-defined lesion (calipers). The lesion blends with the parenchyma without displacing it (arrows), exemplifying the absence of a space-occupying effect. Posterior acoustic shadowing is present. These features are consistent with a BI-RADS 4b assessment. **(C, D)** Mammographic CC **(C)** and MLO **(D)** views reveal focal glandular thickening with increased density in the left breast (BI-RADS 0), with no discrete mass. **(E)** Histopathological examination (H&E stain) shows marked stromal hyalinization and sclerosis with lymphocytic infiltration around breast lobules, diagnostic of diabetic mastopathy.

**Figure 2:**
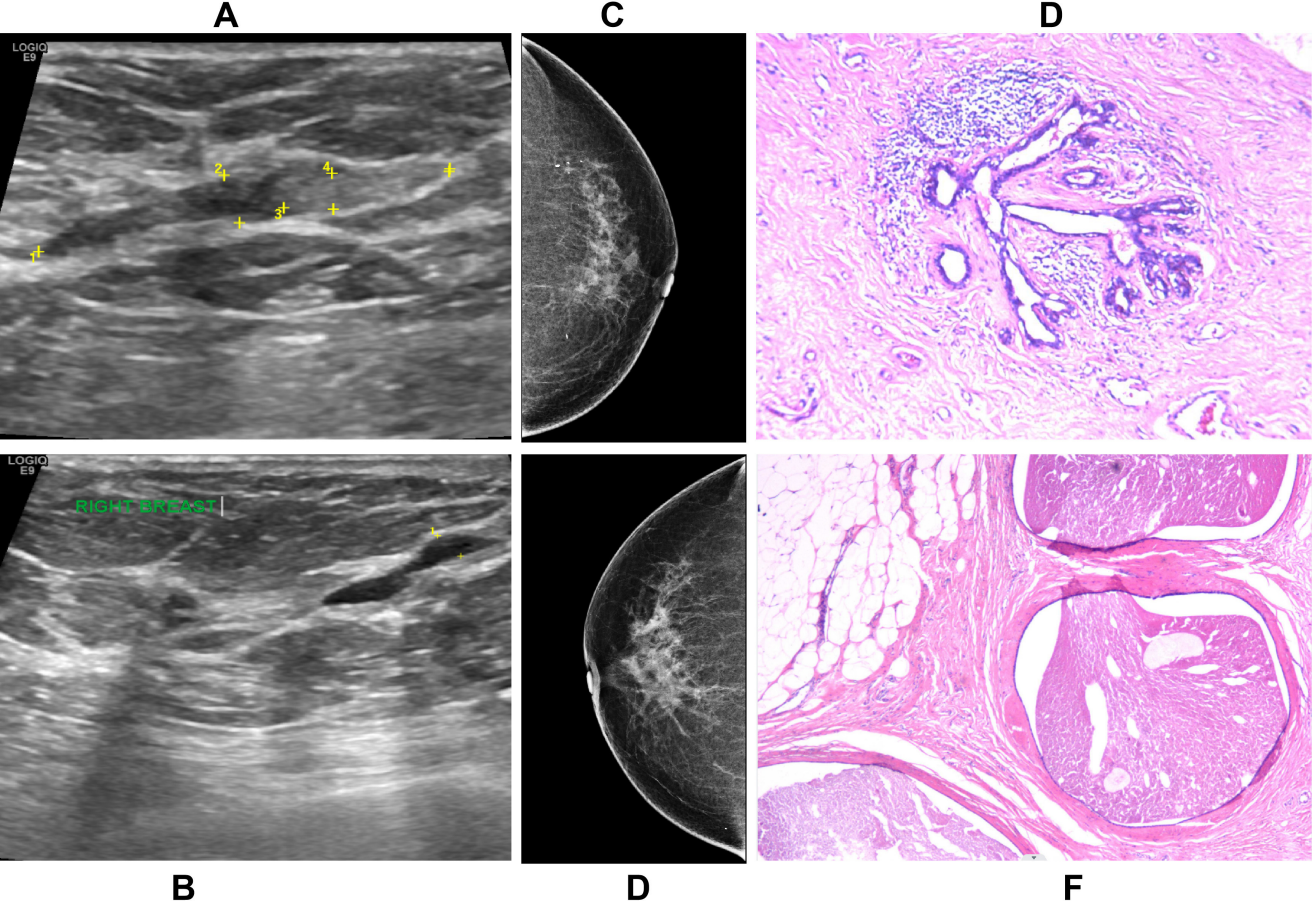
A 51-year-old woman (Patient 17, **Table 3**) with 22-year history of type 2 diabetes mellitus (12 years insulin therapy) presenting with persistent nipple discharge for 9 months. **(A, B)** Ultrasonographic images demonstrate bilateral ductal dilatation, with solid intraductal echogenic material in the left breast **(A)** and echogenic debris in the right breast **(B)**, both classified as BI-RADS 4a. **(C, D)** Mammographic CC views of the left **(C)** and right **(D)** breasts show dense parenchyma without suspicious findings (BI-RADS 3 bilaterally). **(E, F)** Histopathological sections reveal characteristic features of diabetic mastopathy, including lobular atrophy, stromal sclerosis, ductal ectasia with secretory retention **(E)**, and periductal lymphocytic infiltration **(F)**.

In this study, all 21 DMP lesions underwent ultrasonographic examination. Of these, 16 patients (19 lesions) presented with palpable breast masses (**Figure 1**), while one patient (bilateral lesions) presented with nipple discharge as the primary symptom, without a discrete palpable mass (**Figure 2**).

The sonographic characteristics of the 19 mass-forming lesions are synthetically detailed in **Table 2**. The lesions ranged in size from 21 to 65 mm, with a median size of 36 mm, and all were solid masses. All lesions (100%) exhibited irregular margins. Echotexture varied: the majority (78.9%, 15/19) presented as a heterogeneous, ill-defined area that intermingled with and did not distort the surrounding glandular architecture, demonstrating a clear absence of a space-occupying effect. The remaining lesions (21.1%, 4/19) were discrete hypoechoic masses. Posterior acoustic shadowing was a prominent feature, present in 89.5% (17/19) of cases. On Doppler imaging, all lesions were avascular (100%). Coarse calcifications were absent in 94.7% (18/19) of lesions; and similarly, axillary lymphadenopathy was not observed in any case (100%). 

**Table 2:** Ultrasonographic features of 19 mass-forming DMP lesions.

Characteristic	Number (%)
Nature	
Solid	19 (100%)
Lesion type	
Infiltrative heterogeneous area (ill-defined, blending with parenchyma)	15 (78.9%)
Discrete hypoechoic mass	4 (21.1%)
Shape	
Irregular	19 (100%)
Posterior acoustic shadowing	
Present	17 (89.5%)
Absent	2 (10.5%)
Orientation	
Parallel to skin	19 (100%)
Calcifications	
Absent	
Coarse	1 (5.3%)
Vascularity	
Avascular	19 (100%)
Axillary lymphadenopathy	
Absent	19 (100%)

DMP, diabetic mastopathy.

Based on these features, the mass lesions were classified as follows: 14 (73.7%) as BI-RADS 4a, 4 (21.1%) as BI-RADS 4b, and 1 (5.3%) as BI-RADS 5. In the patient with bilateral, non-mass lesions, ultrasonography revealed multiple dilated ducts containing intraductal material, and both were classified as BI-RADS 4a (**Figure 2**). The detailed clinical and imaging characteristics of all 21 DMP lesions are summarized in **Table 3**.

**Table 3:** Clinical and imaging characteristics of 21 DMP lesions.

Patient number	Age (years)	Lesion number	Size (mm)^†^	Lesion type	US BI-RADS	MG BI-RADS
1	68	1	33	Palpable mass	BI-RADS 4b	BI-RADS 0
2	66	2	38	Palpable mass	BI-RADS 4a	—
3	57	3	65	Palpable mass	BI-RADS 4b	BI-RADS 4b
4	50	4	53	Palpable mass	BI-RADS 5	BI-RADS 0
5	62	5	41	Palpable mass	BI-RADS 4a	BI-RADS 3
6	58	6	26	Palpable mass	BI-RADS 4a	BI-RADS 3
7	43	7	36	Palpable mass	BI-RADS 4b	BI-RADS 3
8	58	8	27	Palpable mass	BI-RADS 4b	BI-RADS 3
9	55	9	26	Palpable mass	BI-RADS 4a	BI-RADS 3
10	51	10	60	Palpable mass	BI-RADS 4a	BI-RADS 3
11	66	11	39	Palpable mass	BI-RADS 4a	BI-RADS 3
12	60	12	32	Palpable mass	BI-RADS 4a	BI-RADS 3
13	58	13	63	Palpable mass	BI-RADS 4b	—
14	62	14	30	Palpable mass	BI-RADS 4a	BI-RADS 3
		15	35	Palpable mass	BI-RADS 4a	BI-RADS 3
15	56	16	25	Palpable mass	BI-RADS 4a	BI-RADS 3
		17	27	Palpable mass	BI-RADS 4a	BI-RADS 3
16	55	18	51	Palpable mass	BI-RADS 4a	BI-RADS 3
		19	37	Palpable mass	BI-RADS 4a	BI-RADS 2
17	51	20	—^‡^	Nipple discharge	BI-RADS 4a	BI-RADS 3
		21	—^‡^	Nipple discharge	BI-RADS 4a	BI-RADS 2

^†^ Size, greatest diameter measured on ultrasound.

^‡^ Not applicable for non-mass lesions.

— Not performed, not applicable, or data unavailable.

BI-RADS, Breast Imaging Reporting and Data System; US, ultrasonography; MG, mammography.

### 3.4 Pathologic diagnosis and follow-up

The detailed pathological diagnosis, core needle biopsy correlation, and follow-up data for each lesion are summarized in **Table 4**. All 21 lesions were ultimately confirmed as diabetic mastopathy (DMP) through definitive pathological assessment. Surgical excision served as the diagnostic reference standard for 16 lesions. Among the remaining 5 lesions managed non-operatively, diagnosis was established via concordant clinical, imaging, and core needle biopsy findings.

**Table 4:** Pathological diagnosis and follow-up results of 21 DMP lesions.

Patient	Lesion	CNB Result	Surgical Result	Follow-up (months)	Outcome
1	1	Marked sclerosis	DMP	18.6	No recurrence
2	2	DMP	DMP	20	New lesion
3	3	Sclerosing adenosis	DMP	42	No recurrence
4	4	DMP	DMP	Lost	Lost to follow-up
5	5	DMP	No surgery	74.2	Lesion resolved
6	6	DMP	DMP	35	New lesion
7	7	DMP	DMP	Lost	Lost to follow-up
8	8	Lymphocyte infiltration	DMP	40	No recurrence
9	9	DMP	No surgery	37	Size increased
10	10	DMP	DMP	Lost	Lost to follow-up
11	11	DMP	No surgery	15	Size increased
12	12	DMP	DMP	65	New lesion
13	13	Chronic mastitis	DMP	5	No recurrence
14	14	DMP	DMP	19.5	No recurrence
	15	DMP	DMP	19.5	No recurrence
15	16	DMP	No surgery	23.0	No change
	17	DMP	No surgery	23.0	No change
16	18	Nonspecific fibrosis	DMP	40.6	No recurrence
	19	Nonspecific fibrosis	DMP	40.6	No recurrence
17	20	—	DMP	33.5	No recurrence
	21	—	DMP	33.5	No recurrence

— Not performed.

CNB, core needle biopsy; DMP, diabetic mastopathy.

Ultrasound-guided core needle biopsy (CNB) was performed on 19 lesions. It yielded a specific histological diagnosis of DMP in 13 cases (68.4%). In the remaining 6 cases (31.6%), CNB results were non-specific, reporting fibro-inflammatory changes (e.g., marked sclerosis, chronic inflammation). All 6 lesions with non-specific CNB results underwent surgical excision for definitive diagnosis, contributing to the overall surgical rate.

During a median imaging follow-up of 33.5 months (range: 5–74.2 months) available for 18 lesions, clinical outcomes differed between management groups. Among the 13 surgically treated lesions with follow-up, 10 (76.9%) remained recurrence-free, while 3 (23.1%) developed new ipsilateral lesions radiologically suggestive of DMP, all confirmed by repeat CNB. In the non-surgical group (n=5), two lesions progressed in size, two remained stable, and one resolved spontaneously. Importantly, no breast carcinoma was detected during follow-up in any patient.

## 4 Discussion

### 4.1 Key findings and diagnostic paradox

This multi-center analysis of 21 DMP lesions from 17 patients highlights its diagnostically challenging nature. Key findings include: 1) a notable predominance of type 2 diabetes (64.7%) in our cohort, which may reflect the increasing global prevalence of T2DM and the extended lifespan of patients with chronic diabetes; 2) a characteristic imaging paradox: mammography was predominantly benign (BI-RADS 3), whereas ultrasound revealed suspicious solid masses. Importantly, most (78.9%) of these masses displayed an infiltrative growth pattern with a distinctive “absence of space-occupying effect,” serving as a crucial sonographic clue to distinguish DMP from malignant lesions; 3) identification of an atypical, non−mass presentation featuring ductal dilatation and intraductal debris; and 4) a high surgical intervention rate (76.2%), largely attributable to diagnostic uncertainty despite the benign nature of DMP. The diagnostic accuracy of core−needle biopsy was suboptimal (68.4%), and post−surgical recurrence was observed in 23.1% of cases.

### 4.2 Contextualizing novelty within the existing literature

Our findings corroborate and extend the established understanding of DMP. While the condition has historically been associated with long-standing type 1 diabetes (T1DM) (4, 10), our cohort demonstrated a predominance of type 2 diabetes (T2DM) (64.7%). This discrepancy is not contradictory but may reflect the contemporary epidemiological shift and improved longevity in diabetes care. Given the increased global incidence of type 2 diabetes mellitus (T2DM) (11) and the prolonged survival of patients with long-standing diabetes due to advancements in management (12, 13), T2DM now constitutes a larger pool of patients at risk for chronic complications such as DMP. This supports the hypothesis that the pathogenesis of DMP is more closely linked to chronic hyperglycemia and its autoimmune sequelae than to the specific diabetes type (14, 15).

The classic imaging paradox—benign mammographic findings alongside suspicious sonographic masses—is well-documented (14, 15). Beyond confirming known features such as irregular margins and posterior shadowing, a key contribution of this study is the systematic evaluation of a predominant sonographic pattern observed in 78.9% of mass-forming lesions: an infiltrative heterogeneous area with ill-defined margins that seamlessly blends with the surrounding parenchymal architecture. We define this pattern as the direct imaging correlate of the “absence of a space-occupying effect,” characterized by the lack of displacement or architectural distortion of normal tissue. Pathologically, this sign is grounded in the diffusely infiltrative stromal fibrosis characteristic of DMP (16, 17), which permeates rather than displaces normal tissue. This stands in contrast to the expansile growth of breast carcinoma, which typically presents as a discrete mass causing architectural distortion. Thus, the “absence of a space-occupying effect” provides a critical differential diagnostic clue. While previous literature (15, 18) has sporadically noted that DMP lesions may be “ill-defined,” this study is among the first to clearly delineate and operationalize this specific sign as a distinct, observable, and highly suggestive imaging feature, providing radiologists with a practical pre-biopsy clue that may help reduce unnecessary interventions.

Furthermore, the atypical presentation of DMP as ductal dilatation with intraductal debris, which we report, expands the known sonographic spectrum of this disease. While atypical presentations such as clustered microcalcifications (19) and diffuse hyperechogenicity (20) on ultrasound without discrete masses have been documented in isolated case reports, this specific duct-centric pattern has not, to our knowledge, been previously described.

The primary differential for mass-forming DMP is breast cancer (21, 22). Key distinctions include clinical history (long-standing diabetes *vs*. family history of cancer) (23, 24), sonographic features (infiltrative growth without mass effect *vs*. space-occupying hypoechoic masses) (25, 26), and imaging findings (rare calcifications and typically negative mammography in DMP *vs*. frequent calcifications and positive mammography in cancer) (27). Pathological confirmation remains paramount, noting the established limitation of CNB in DMP due to its patchy fibrosis, which often yields non-specific results and may lead to surgical excision for diagnosis.

Beyond ultrasound and mammography, breast magnetic resonance imaging (MRI) plays an important role in the diagnostic workup of ambiguous breast lesions. Recent literature (10) has delineated characteristic MRI features of DMP, including persistent (Type I) enhancement kinetics and non-mass-like enhancement, which help differentiate it from breast cancer. In clinical practice, MRI may be particularly useful in specific scenarios, such as cases with imaging–pathology discordance after core needle biopsy, persistently equivocal or suspicious findings on ultrasound), or when evaluating the extent of suspected infiltrative lesions. Therefore, in the diagnostic workup of DMP, MRI can be best viewed as a complementary problem-solving tool rather than a first-line modality. Its value lies in aiding critical management decisions—such as choosing between conservative follow-up and proceeding to targeted biopsy or surgical planning—in complex or equivocal cases. Integrating MRI in these situations can provide valuable adjunctive information to guide further management decisions.

The diagnostic challenge of DMP extends to its histopathologic confirmation. The suboptimal accuracy of ultrasound-guided core needle biopsy (CNB) in our series—yielding a specific diagnosis in only 68.4% (13/19) of lesions—aligns with and quantifies a well-documented hurdle in the literature (14, 15). Prior studies (28) have similarly reported frequent non-specific results, often described as “fibrosis” or “chronic inflammation,” due to the patchy and infiltrative nature of the disease which eludes definitive sampling by percutaneous needles. This inherent limitation has significant management implications. In our cohort, diagnostic uncertainty was a direct driver of intervention, as all lesions with non-specific CNB results proceeded to surgical excision. This pattern reinforces the established clinical pathway where excisional biopsy often remains necessary for definitive diagnosis, despite initial percutaneous sampling (29, 30).

This diagnostic ambiguity necessitates a clear understanding of the relationship between DMP and breast cancer risk. It is well-established that DMP is a benign, non-premaliant entity, and its histologic features are not considered direct precursors to malignancy (14, 15). The absence of breast cancer in our cohort aligns with this consensus. However, it is crucial to recognize that patients with long-standing diabetes (the population at risk for DMP) may have independent risk factors for breast cancer, and the two conditions can co-exist. As highlighted by Mariano et al. (10), breast cancer can occur independently in patients with DMP. Therefore, the clinical focus should be on differentiation and co-detection rather than implying a causal link. A dual management strategy is recommended (1): a thorough initial diagnostic workup, including pathological verification, to reliably distinguish DMP from carcinoma and rule out concurrent malignancy; and (2) adherence by patients with DMP to standard, population-based breast cancer screening protocols, as their underlying risk profile warrants continued surveillance.

### 4.3 Clinical implications and integration into practice

This study highlights key considerations for managing DMP. Clinicians should suspect DMP in patients with long-standing diabetes (both type 1 and type 2) and a firm breast mass, particularly given the expanding population of patients with long-duration type 2 diabetes. Radiologists can use the “absence of a space-occupying effect” as a specific clue for DMP when assessing solid masses in diabetic patients, aiding biopsy decisions. Awareness of the ductal dilatation pattern is crucial to avoid misdiagnosis in cases with nipple discharge. Given the limited accuracy of core needle biopsy, nonspecific results in suggestive cases should prompt consideration of DMP, possibly favoring repeat biopsy or surveillance over immediate surgery, especially considering recurrence risks. Overall, a conservative, multidisciplinary approach is recommended.

### 4.4 Study limitations

This study has several limitations. The retrospective, multi-center design may introduce selection bias and data heterogeneity. Excluding cases with only descriptive pathology reports could have omitted atypical presentations. The small sample size limits statistical power and generalizability, particularly for rare findings like the ductal pattern. The absence of a standardized MRI protocol represents a significant methodological gap, preventing us from evaluating its potential to improve diagnostic accuracy in our cohort. Finally, variability in ultrasound equipment and the absence of formal inter-reader agreement assessment may affect reproducibility.

### 4.5 Future research directions

Future prospective, multi-center studies with standardized imaging (including MRI) are needed to validate our findings, especially the “absence of space-occupying effect” and ductal patterns, in larger cohorts. Research should establish evidence-based imaging criteria to improve pre-biopsy diagnosis. Investigating links between glycemic control, diabetic complications, and DMP phenotype could clarify pathogenesis. Large-scale, long-term cohorts are essential to define any relationship between DMP and breast cancer risk, guiding surveillance strategies.

## Data Availability

The raw data supporting the conclusions of this article will be made available by the authors, without undue reservation.
